# The chemosensory world of mosquitoes: olfactory receptors and their role in blocking mosquito-borne disease transmission

**DOI:** 10.1186/s13071-025-06954-1

**Published:** 2025-08-02

**Authors:** Sitian Xiong, Jingjing Liang, Shuyang Gao, Zhilong Liu, Hong Zheng, Xuesen Yang, Ying Wang, Shasha Yu

**Affiliations:** 1 Department of Tropical Medicine, College of Military Preventive Medicine, Army Medical University, No. 30 Gaotanyan Street, Shapingba District, Chongqing, 400038 China; 2 Department of Thoracic Surgery, Xinqiao Hospital, Army Medical University, Chongqing, 400037 China

**Keywords:** Olfactory receptors, Mosquito-borne diseases, Repellents, Vector control

## Abstract

Mosquitoes are vectors for a variety of infectious diseases, which pose a significant threat to human health, public well-being, and national security. The increasing prevalence of insecticide resistance has further complicated efforts to control these diseases, highlighting the urgent need for innovative strategies to block their transmission. The mosquito olfactory system, which mediates a wide range of behaviors throughout the mosquito's life cycle, including host-seeking, foraging, oviposition, mating, avoidance behavior, and social communication, offers a promising avenue for intervention. Specifically, olfactory receptors play a crucial role in enabling female mosquitoes to locate human hosts through odor recognition, a process that facilitates the transmission of pathogens responsible for diseases such as malaria and dengue fever. Understanding the roles of these receptors in different mosquito behaviors, their mechanisms of odor recognition, and their involvement in disease transmission could provide valuable insights for developing new mosquito control strategies that target olfactory receptors to effectively block disease spread. This review aims to provide a comprehensive summary of the mosquito olfactory system, including its composition, the mechanisms of action of olfactory receptors, their interactions with repellents, and their potential role in the prevention and control of mosquito-borne diseases.

**Graphical Abstract:**

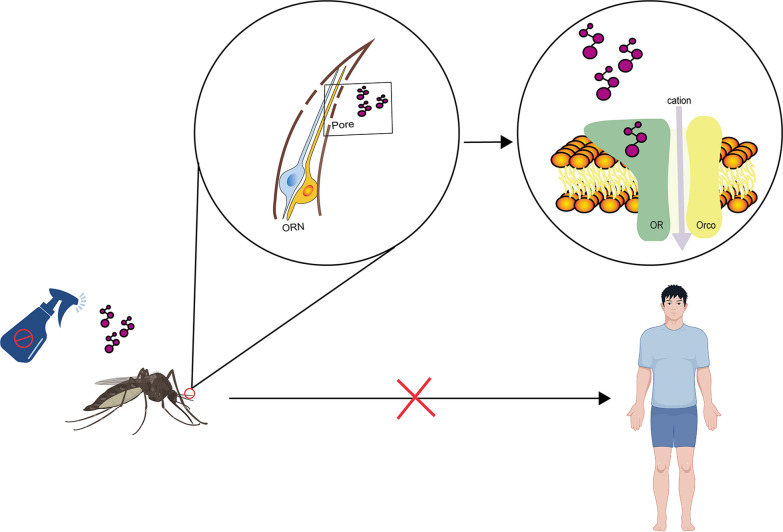

## Background

Mosquitoes, particularly females, are significant vectors for numerous pathogens, transmitting a wide range of diseases, including malaria, dengue fever, Japanese encephalitis, yellow fever, and filariasis [1]. These diseases pose a severe threat to human health, especially in tropical and subtropical regions [2–4]. According to the World Health Organization, an estimated 249 million malaria cases occurred worldwide in 2022, marking an increase of 16 million from pre-COVID-19 levels [5]. Additionally, the global incidence of dengue fever has risen sharply in recent decades, with approximately 390 million annual infections [6, 7]. The risk of yellow fever in Asia has also increased due to its introduction to China following the 2016 Angola outbreak and continued globalization [8].

Among the current mosquito-borne disease control measures, long-lasting insecticidal nets (LLINs) and indoor residual spraying (IRS) are the most widely adopted [9, 10]. However, the growing prevalence of insecticide resistance and the emergence of new arthropod-borne viruses highlight the urgent need for novel approaches [11]. Recent biological methods offer a more sustainable approach to mosquito control, including the use of genetically modified mosquitoes. Genetic editing technologies, such as clustered regularly interspaced short palindromic repeats (CRISPR)/CRISPR-associated protein 9 (Cas9), have shown promise in developing mosquitoes that are less capable of transmitting diseases [12]. However, the use of genetically modified mosquitoes for vector-borne disease control is still in its infancy, and implementing these measures requires time and effort, as many communities are strongly concerned about potential negative impacts [13, 14]. One promising strategy is to disrupt mosquito survival and reproduction by interfering with their olfactory system, which is crucial for detecting food sources, mating, and oviposition sites. Research on mosquito olfactory mechanisms can also aid in developing repellents and attractants to prevent blood-feeding and trap mosquitoes, respectively.

Mosquitoes inhabit diverse environments and have evolved sophisticated sensory systems to detect complex environmental stimuli and biological cues. Throughout their adult life stages, mosquitoes predominantly rely on their olfactory system to locate nectar sources [15]. During mating, mosquito spermatozoa express odorant receptors (ORs), which may activate dormant sperm and exert chemotactic functions [16]. After fertilization, female mosquitoes use their olfactory system to find hosts for blood meals, which provide essential proteins and nutrients for egg development [17]. Additionally, interactions among females in oviposition site selection and optimal densities are also mediated by olfaction [18–20].

Given the pivotal role of olfactory receptors in mosquito survival and reproduction, this review delves into recent research on mosquito olfactory receptors and explores their potential for preventing and controlling mosquito-borne diseases. A comprehensive understanding of these receptors may unveil novel targets for curbing mosquito populations and mitigating disease transmission.

## Mosquito olfactory system

**Fig. 1:**
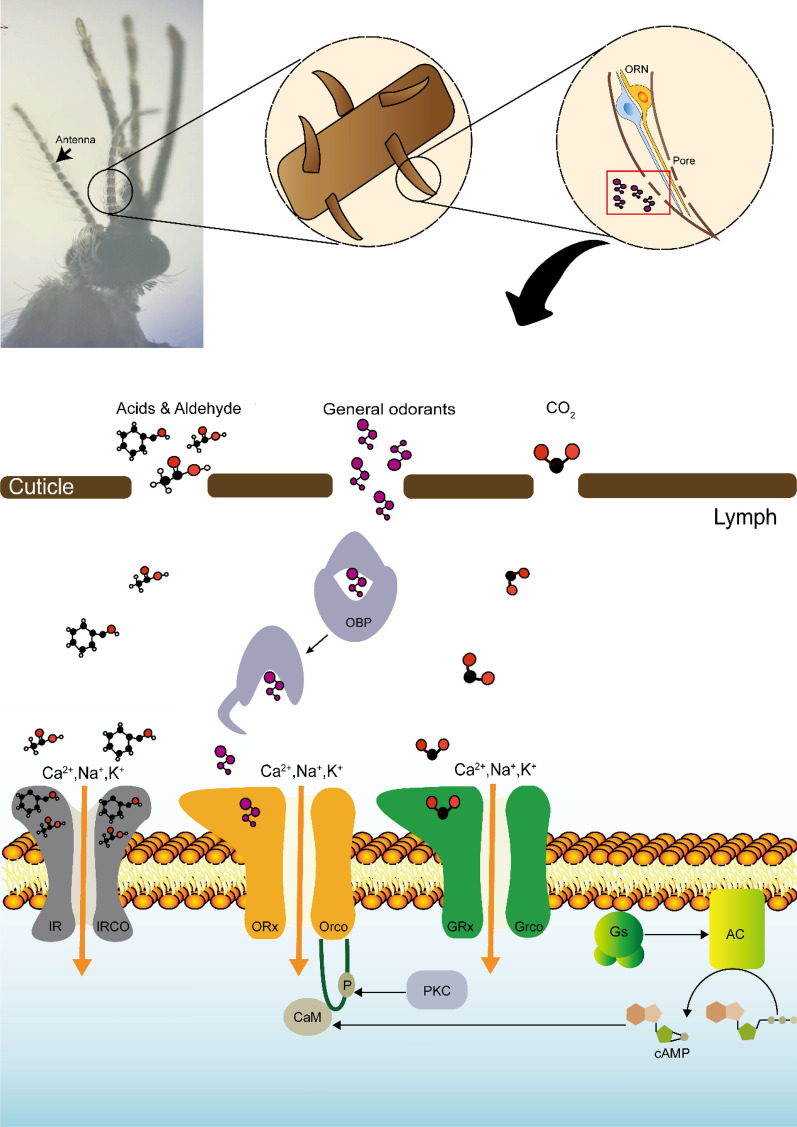
The mosquito peripheral olfactory system detects chemical volatiles via olfactory receptor neurons (ORNs). During olfactory transduction, odor molecules enter the lymph through pores in the olfactory epithelium, interact with odorant-binding proteins (OBPs), and are transported to olfactory receptors. These receptors form heterodimers with co-receptors, creating ion channels in ORN dendrites. Upon binding, ion channels open, allowing cations to flow into the cell and activate the neuron. The ORx subunit also activates a G protein, which enhances adenylate cyclase (AC) activity and increases cyclic adenosine monophosphate (cAMP) production, further activating ORx/odorant receptor co-receptor (Orco) channels. The Orco activity is regulated by calmodulin (CaM) or protein kinase C (PKC) phosphorylation

Mosquitoes detect chemical volatiles through three specialized head appendages: the antennae, maxillary palps, and proboscis labella. The antennae, densely covered with sensilla, primarily house the olfactory receptors that detect volatile compounds. The labella, also equipped with sensilla, are vital for close-range host attraction. These structures are covered with specialized sensory hairs known as sensilla, each typically containing two to three olfactory receptor neurons (ORNs) [21, 22] (Fig. 1). ORN axons from the antennae and maxillary palps project to the antennal lobe (AL), which is usually spheroidal and further divided into smaller neuropils called glomeruli [23, 24]. These axons relay information to higher brain centers, namely the mushroom body and the lateral horn, via projection neurons (PNs). Meanwhile, local neurons (LNs) provide lateral connections which encode olfactory information into a spatial odor map for further processing in higher brain areas [25]. In contrast, ORN axons from the proboscis labella project to the suboesophageal zone [26].

Olfactory receptors are expressed in ORNs. Traditionally, the “one neuron–one receptor” and “one glomerulus–one receptor” principles were believed to govern mosquito olfactory system organization, meaning that each olfactory sensory neuron expressed only a single olfactory receptor type, and all neurons expressing the same receptor projected their axons to the same region [27]. However, recent studies reveal that mosquitoes co-express multiple chemosensory receptors in a single neuron, enhancing their olfactory sensitivity and complicating efforts to disrupt their human-detection abilities. For example, maxillary palp capitate peg sensilla contain CO_2_-responsive neurons (cpA) expressing CO_2_-sensing gustatory receptors (GRs), as well as neurons expressing ORs. Antennal sensilla house ORNs expressing ORs, ionotropic receptors (IRs), or both [28]. This co-expression of receptors underscores the adaptability and robustness of the mosquito olfactory system, posing both challenges and opportunities for developing novel vector control strategies.

## Olfactory receptor families

**Fig. 2:**
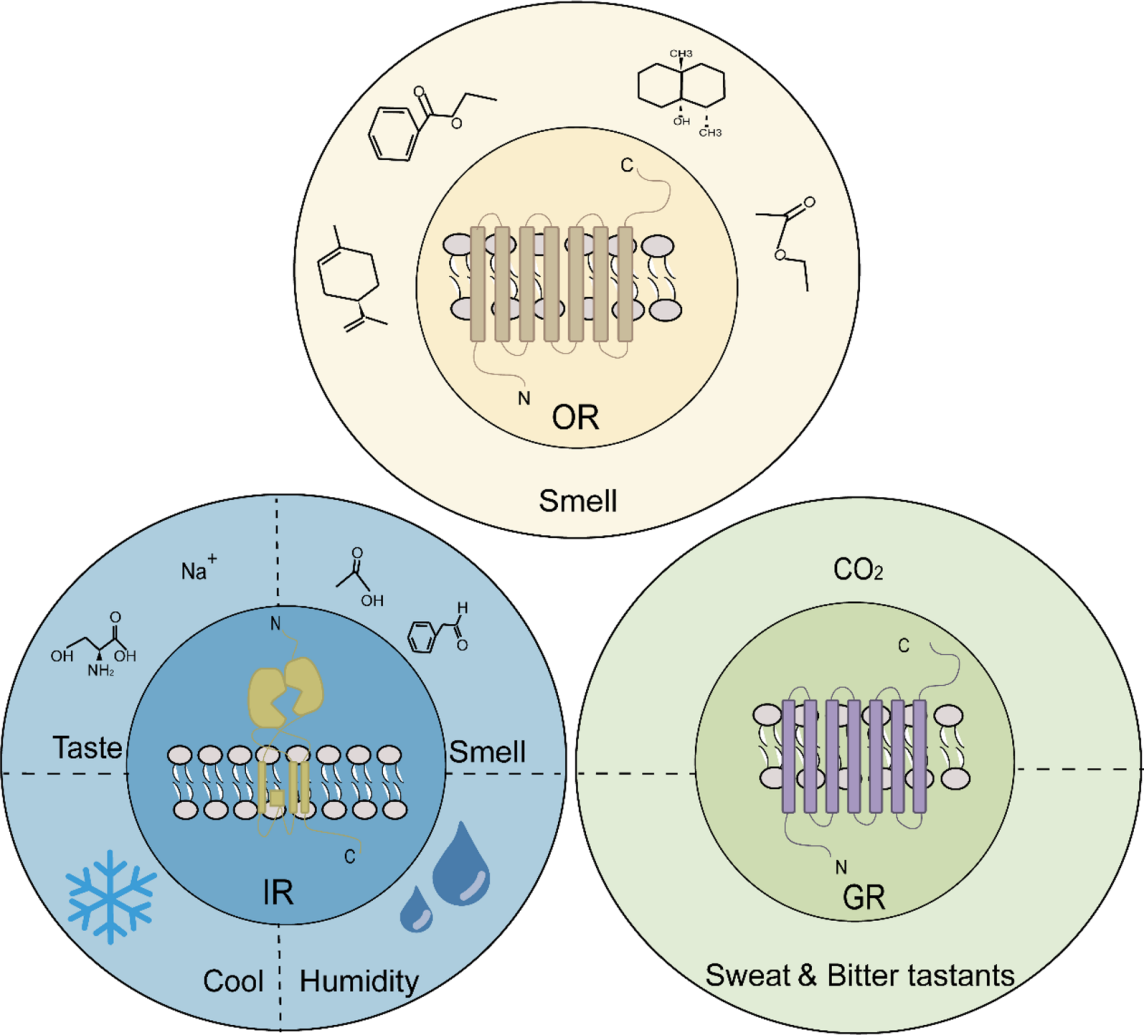
Mosquito olfactory receptors are categorized into three families: gustatory receptors (GRs), ionotropic receptors (IRs), and odorant receptors (ORs). ORs primarily detect volatile chemical signals, while IRs sense tastes, humidity, and temperature in addition to volatile compounds. GRs are capable of detecting taste modalities such as sweetness and bitterness, and are also highly sensitive to CO_2_

There are three main classes of receptors involved in the olfactory families: GRs, IRs, and ORs (Fig. 2). Each class has a distinct structure and plays a different role in the olfactory system [29]. Mosquito ORs can be divided into two categories: highly divergent ligand-binding ORs and highly conserved odorant receptor co-receptor (Orco) [30, 31]. ORs represent one of the largest ion channel families in the animal kingdom, with highly divergent sequences across numerous insect species. To date, 59 OR genes have been identified in *Anopheles sinensis* [32], 180 in *Culex quinquefasciatus* [33], 79 in *Anopheles gambiae* [34], 131 in *Aedes aegypti* [35], and 82 in *Aedes albopictus* [36]. Despite the large number of genes, only a small fraction have been functionally characterized, highlighting the potential for discovering new molecular targets for mosquito control [37, 38].

IRs, a novel class of olfactory receptors associated with ionotropic glutamate receptors, share similar molecular structures despite lacking amino acid homology [39, 40]. A functional IR complex comprises a specific ligand-sensing IR and a co-receptor (IR25a or IR8a), both expressed in antennal neurons [41, 42]. The odor response spectrum of olfactory sensory neurons expressing IRs overlaps with that of neurons expressing ORs, thereby enabling the mosquito olfactory system to detect a broader range of chemical signals [43].

GRs, initially identified on fruit fly labella for detecting sweet and bitter substances, are also widely expressed in mosquitoes [44, 45]. These receptors share a similar structure with ORs, featuring seven transmembrane domains with an intracellular N-terminus and an extracellular C-terminus. GRs form tetrameric sugar-gated cation channels, composed of a central pore domain and four peripheral ligand-binding domains [46]. In mosquitoes, CO_2_-sensitive GRs are crucial for host-seeking, as blood-feeding females locate hosts through exhaled CO_2_ [47, 48]. Beyond CO_2_ detection, certain GRs are involved in pheromone detection and oviposition, playing an essential role in mosquito reproductive behaviors [49, 50]. The absence of these receptor subtypes may result in reduced mating efficiency in males [51].

## Mechanism of action of olfactory receptors

### Selectivity of olfactory receptors

Odors consist of millions of molecules that vary widely in shape, size, and chemical properties. Mosquitoes rely on their olfactory system to detect these diverse molecules using a limited number of ORs to accomplish various physiological functions [52]. To achieve this, the mosquito olfactory system has evolved a combinatorial coding mechanism, where specific odor molecules can activate multiple ORs, and each OR can respond to multiple odorants. This allows mosquitoes to recognize a vast array of odor molecules with a relatively small number of receptors [25]. For example, ablating some OR genes does not affect mosquito behavior, indicating redundancy in the system [53]. However, OR expression varies by mosquito sex [32], age [54], and behavior [55–57], suggesting that different ORs may have specialized roles beyond the combinatorial coding pattern. Detailed studies on individual ORs expressed in heterologous systems reveal that some ORs are narrowly tuned to a single odorant, while others are more broadly tuned [58–60].

OR8 is highly conserved across mosquito species, including *An. gambiae*, *Ae. aegypti*, *Cx. quinquefasciatus*, and the non-blood-feeding elephant mosquito *Toxorhynchites amboinensis* [61]. In *An. gambiae*, AgOR8 specifically recognizes 1-octen-3-ol [62], a common human odor that attracts mosquitoes when combined with CO_2_ [63, 64]. Similarly, in *Ae. aegypti*, AaOR8 responds specifically to the (*R*)-enantiomer of 1-octen-3-ol at genetic [65], electrophysiological [66], and behavioral levels [67]. In *Cx. quinquefasciatus*, reducing the expression of CquiOR114/117 (a homolog of AaOR8) impairs blood-feeding behavior [68], while 1-octen-3-ol binding to CquiOR32 produces inhibitory responses [69].

OR2 and OR10 are also highly conserved and expressed in *Cx. quinquefasciatus* [70, 71], *Ae. aegypti* [72], and *An. gambiae* [73, 74]. These receptors detect indole and skatole, which regulate oviposition and host location behavior [75, 76]. Studies have shown that a single amino acid change in OR2 and OR10 can reverse the specificity of these receptors for these odorants [77], suggesting the possibility of designing effective oviposition attractants to trap mosquitoes. Additionally, AaOR4 in *Ae. aegypti* detects human-specific odor compounds like sulcatone, enabling mosquitoes to prefer humans over other animals [78].

In summary, while some odor molecules can activate multiple ORs and vice versa, certain receptors exhibit high specificity for particular compounds, including host attractants and repellents (Table 1). This specificity is crucial for minimizing cross-activation of ecologically relevant compounds and provides potential targets for controlling mosquito behavior and interrupting disease transmission.

**Table 1:** Mosquito olfactory receptors specifically activated by repellents and other odor molecules

Compound	Mosquito species	Odorant receptor	References
1-Octen-3-ol	*An. gambiae*	OR8	[62]
	*Ae. aegypti*	OR8	[65, 67]
	*Cx. quinquefasciatus*	OR114/117, OR32	[68]
	*Tx. amboinensis*	OR8	[61]
Indole and skatole	*An. gambiae*	OR2/OR10	[73, 74]
	*Ae. aegypti*		[72]
	*Cx. quinquefasciatus*		[70, 71]
Sulcatone	*Ae. aegypti*	OR4	[55, 78]
4-Chromanone	*Ae. aegypti*	OR6	[59]
Borneol	*Tx. amboinensis*	OR49	[79]
	*Ae. aegypti*	OR49	[79]
	*Cx. quinquefasciatus*	OR38	[79]
	*Ae. albopictus*	OR49	[79]
Fenchone	*Ae. aegypti*	OR11	[80]
	*Ae. albopictus*	OR11	[81]
Geranyl acetate and (E)-β-farnesene	*Ae. aegypti*	OR31	[82, 83]
Linalool	*An. gambiae*	OR29	[84]
	*An. stephensi*	OR53	[84]
	*Cx. quinquefasciatus*	OR5	[85]
Methyl salicylate	*Cx. quinquefasciatus*	OR32	[69]
Methyl dihydrojasmonate	*Cx. quinquefasciatus*	OR136	[86, 87]
2-Phenylethanol	*Cx. quinquefasciatus*	OR4	[85]
Acetophenone	*Ae. aegypti*	OR15	[85]
Phenethyl formate, phenethyl propionate	*Cx. quinquefasciatus*	OR4	[85]
	*Ae. aegypti*	OR15	[85]

### Structure and functions of olfactory receptors

Understanding the structure of ORs is crucial for elucidating how the olfactory system recognizes and discriminates an almost unlimited number of chemical cues in the environment. The OR gene family in insects has significantly expanded over evolutionary time, yet key structural features and binding-site residues are conserved across different subfamilies, suggesting a certain universality to OR structures across various insect species [37, 38].

The first insect ORs were identified several decades ago, and it took another 7 years to achieve the first two-dimensional representation of ORs with correct transmembrane topology [88, 89]. ORs and Orco are seven-transmembrane proteins, with an intracellular N-terminus and an extracellular C-terminus, displaying inverted topology compared to their vertebrate and nematode counterparts [90–92]. Significant insights have been gained through site-directed mutagenesis, resonance energy transfer, and structural modeling [93–95]. For instance, the second extracellular loop of ORs forms a lid over the binding site, and certain extracellular regions of the transmembrane helices are involved in ligand recognition and receptor tuning. Additionally, potential phosphorylation sites and calmodulin (CaM)-binding sites in the second intracellular loop of Orco may regulate channel gating [96], while amino acid substitutions in the third intracellular loop affect Orco’s response speed and sensitivity to repellents [97]. Further research has identified key amino acids involved in the dynamic entry pathway and binding of repellents to Orco in *Drosophila melanogaster*, pinpointing the exact location of the agonist binding site [98].

Recent advances in single-particle cryo-electron microscopy have revealed the intricate structure of Orco, demonstrating that each subunit consists of seven membrane-spanning helical segments (S1–S7), with an intracellular amino terminus and an extracellular carboxyl terminus. Orco assembles into a tetrameric channel structure with four subunits symmetrically arranged around a central pore. The subunits work in seamless coordination to control the central ion conduction channel, with each subunit contributing a single helix (transmembrane segment S7b) to the pore, surrounded by four other helices (S1–S6) that maintain a loose association [99]. The OR complex, comprising specific receptors and Orco, forms a functional unit in a 1:3 stoichiometry, where odorant binding to the OR subunit induces a conformational change that opens the ion channel [100].

The combinatorial response of individual OR ensembles underpins olfactory perception, allowing most ORs to detect a wide array of structurally and chemically diverse odorants. Cryo-electron microscopy studies have unveiled the structural basis of this adaptable chemical detection [99]. However, Orco lacks a ligand-binding site for the receptor, complicating the resolution of the binding site. To circumvent challenges associated with heteromultimeric proteins, researchers have explored the structure of receptors in jumping bristletails (*Machilis hrabei*), whose repertoire includes only five subunits (MhOR1-5), none directly related to Orco. The structure of MhOR5, both individually and in complex with two compounds, shows that odorants are recognized through distributed hydrophobic interactions within a simple binding pocket in the transmembrane region. Notably, weak, non-specific chemical bonds between amino acids and odorants enable the receptor to recognize a broader range of molecules [101]. Accurate modeling of protein–ligand interactions is vital for designing structure-based drugs and repellents.

### Chemosensory transduction in olfactory receptors

Odor detection in the mammalian olfactory epithelium is mediated by ORs, which are G protein-coupled receptors (GPCRs) whose transduction relies on the cyclic adenosine monophosphate (cAMP) second messenger pathway [102, 103]. Therefore, our view of olfactory signal transduction has been somewhat GPCR-centric. Insect ORs differ substantially from mammalian GPCRs. Genetically, mosquito ORs show no sequence similarity or evolutionary relationship with known GPCRs [104]. Insect ORs form heteromultimers comprising a divergent OR subunit, which confers odor specificity, and the highly conserved Orco, which forms the ion channel but does not participate in odor recognition [105, 106]. Orco can create a non-selective cation channel, and can be activated by the synthetic agonist [107, 108]. As Orco is essential for olfactory transduction, its absence impairs olfactory function [109–111].

However, some studies have reported that elements of the G protein transduction pathway are also expressed in insect olfactory neurons, and increases in cAMP concentration can activate ORs and enhance neuronal firing [112–114]. cAMP activation of the OR pathway involves phospholipase C or protein kinase C (PKC), leading to the phosphorylation of amino acid residues on Orco [115, 116]. CaM also stimulates Orco and regulates its function, with mutations in the CaM-binding site on Orco’s second intracellular loop significantly reducing neuronal activity in response to odorants [117].

In the classical olfactory transduction process, odor molecules enter the hydrophilic lymph through the pores in the olfactory epithelium, where they interact with odorant-binding proteins (OBPs). OBPs transport the odor molecules to ORs via aqueous lymph, where they bind to the OR complex, open the ion channel, and allow cations to flow into the cell and activate the neuron [105]. However, insect ORs exhibit additional complexities. First, ORs can form ion channels, providing a fast-acting pathway that activates immediately upon odorant binding. Second, OR signal transduction can also occur through a slower metabolic pathway that regulates Orco after odorant binding.

In summary, the unique structural and functional properties of insect ORs and their distinct transduction pathways highlight the complexity of mosquito olfaction. Understanding these mechanisms not only deepens our knowledge of mosquito behavior but also offers promising avenues for developing novel strategies to disrupt their olfactory systems, thereby reducing mosquito-borne disease transmission and improving public health outcomes.

## Mosquito behavior modulation via receptor–repellent interaction

Modulating mosquito behavior through receptor–repellent interactions is a pivotal strategy in the ongoing battle against mosquito-borne diseases. Historically, early repellants were derived from plant extracts, such as citronella oil, cinnamon oil, and lemon juice. Advances in chemistry have since led to the development of synthetic repellents, including dimethyl phthalate (1929), butoxypropyl di-*n*-propylacetate (1937), and diethyl toluamide (DEET, 1939). More recently, additional synthetic repellents like IR3535 and picaridin have been introduced. These compounds primarily function by disrupting mosquito olfaction, inducing repellent behaviors through activation of ORs or by acting as inverse agonists to inhibit receptor function [87]. The ability to combine repellents further enhances their efficacy [82]. Continued exploration of novel repellents and their mechanisms of action is essential for advancing vector control measures.

### Mechanism of action of DEET

DEET (*N*,*N*-diethyl-3-methylbenzamide) is the most widely used synthetic mosquito repellent. Despite extensive research, its precise mechanism of action remains incompletely understood. DEET appears to function in at least two ways: first, it interacts with chemicals on the skin to reduce the concentration of human odor molecules in the air, thereby hindering mosquito detection; and second, it directly affects mosquito ORs. Specifically, DEET can trigger dose-dependent outward currents that inhibit ORs sensitive to oviposition attractants, disrupting the mosquito’s olfactory system and reducing their sensitivity to human odors [87, 118]. Studies utilizing CquiOR136 have demonstrated that reducing its transcript levels significantly diminishes electroantennographic responses to DEET and eliminates repellency, suggesting that DEET’s efficacy may be linked to its ability to activate mosquito ORs [86]. Additionally, research has confirmed that mosquitoes detect DEET via contact sensors on their legs, reinforcing its role in olfactory disruption [119]. These findings highlight DEET's multifaceted impact on mosquito behavior and olfaction, underscoring its effectiveness as a repellent.

### Activation of olfactory neurons by repellents induces avoidance behavior

The mosquito olfactory system is integral to various behaviors, including foraging, mating, oviposition, habitat selection, and avoidance. Recent studies have shown that specific compounds can activate olfactory neurons, prompting avoidance behaviors. For instance, AaegOR11, one of the most highly expressed ORs in the antennae of female *Ae. aegypti* mosquitoes, responds robustly to compounds such as fenchone, 2,3-dimethylphenol, 3,4-dimethylphenol, 4-methylcyclohexanol, and phenylacetone [120]. Notably, 2,3-dimethylphenol exhibits stronger repellent activity than DEET at equivalent doses [80]. Similarly, AalbOR11 in *Ae. albopictus* can be activated by a diverse array of compounds, including 10 types of aromatics, seven terpenes, six heterocyclic compounds, and three alcohols, with fenchone being the most potent ligand. Fenchone, derived from the essential oil of *Tetradenia riparia*, not only repels mosquitoes but also inhibits acetylcholinesterase, showcasing dual insecticidal and repellent properties. Reducing AalbOR11 expression diminishes the repellent effect of fenchone at low concentrations (0.01% v/v), highlighting OR11's role as a key sensor for repellents and its capacity to trigger avoidance behaviors in mosquitoes [81]. Additionally, AalbOR10, a conserved female-biased OR in *Ae. albopictus*, can be activated by seven aromatic compounds that inhibit egg-laying in blood-fed mosquitoes, including 2-ethylphenol, 2-methylphenol, 3-methylphenol, 4-methylphenol, 3-methylindole, acetophenone, and benzaldehyde [121].

Beyond these specific ORs, other classes of compounds also play significant roles in modulating mosquito behavior. Pyrethroids, a class of insecticides that target voltage-gated sodium channels, exhibit repellent properties [122]. Bio-allethrin (also known as D-trans-allethrin) is a partially decomposed mixture of allethrin isomers, containing two of the eight stereoisomers of allethrin. This commercial pyrethroid, introduced as a mixture of eight stereoisomers, is a common active ingredient in mosquito coils, sprays, evaporative pads, and other mosquito repellent devices. Valbon et al. discovered that bio-allethrin specifically activates neurons of the sst-1 receptor on the antennae of *Ae. aegypti*, leading to spatial repellency [123]. New research indicates that the repellent effect of bio-allethrin is significantly reduced in Orco-mutant mosquitoes, where Orco is an essential obligate olfactory co-receptor for OR-mediated odorant detection that forms odor-gated cation channels with ligand-selective ORs. These findings suggest that bio-allethrin’s repellent action may result from the co-activation of ORs and sodium channels. This dual-target mechanism not only elucidates the mode of action of volatile pyrethroids in spatial repellency but also provides a foundation for developing new repellents [83].

### Suppression of olfactory receptor activity by repellents

Repellents not only induce avoidance behaviors by activating olfactory neurons but also interfere with the olfactory system by inhibiting the activity of olfactory receptors. Many repellents mask and interfere with the mosquito's olfactory system by diminishing the electrophysiological response of neurons elicited by ligands acting on olfactory receptors. For example, recent research has demonstrated that two novel repellents reversibly inhibited odorant-evoked currents from AgOR2/AgOrco and AgOR8/AgOrco receptors in a dose-dependent manner [124]. Octenol, a volatile compound that synergizes with CO_2_ to induce host-seeking behavior in mosquitoes, activates OR8. DEET and indole, both containing aromatic rings and adjacent nitrogen atoms, can interfere with OR8 function. Experimental evidence confirms that indole inhibits the depolarizing current generated by OR8 activation via (R)-1-octen-3-ol, suppressing host-seeking behavior in mosquitoes [125].

**Fig. 3:**
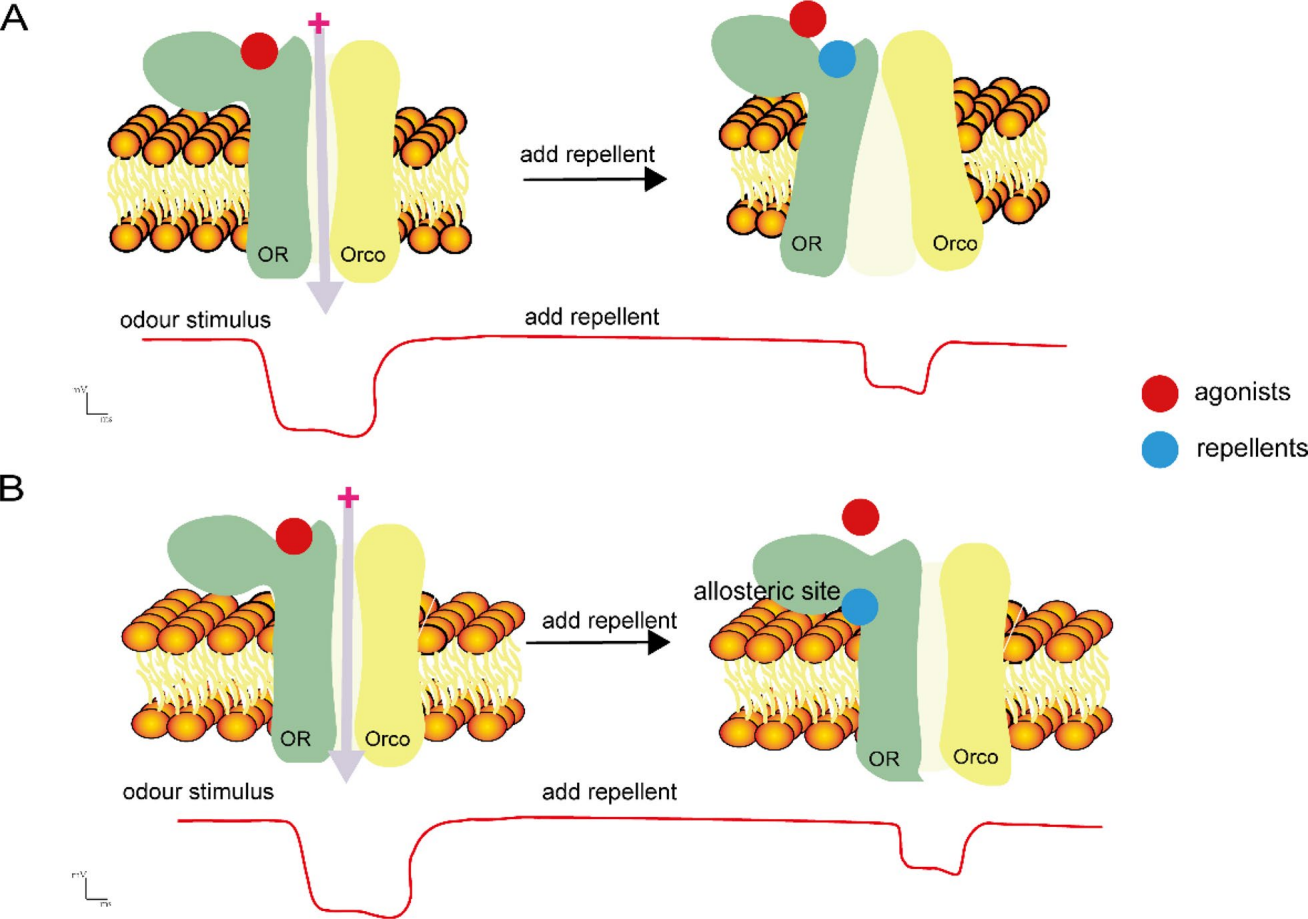
Mechanism of interaction between repellents and olfactory receptors. Repellents bind to a receptor, affecting the receptor’s conductivity and its response to ligands. There are two possible mechanisms: **A** the repellent shifts the receptor’s equilibrium toward a closed state, and **B** the repellent binds to an allosteric site, reducing the receptor’s affinity for the agonist

CquiOR32 acts as an “inhibitory receptor,” generating inhibitory currents when stimulated by eucalyptol. When combined with methyl salicylate, which has repellent properties, eucalyptol can reduce the repellent effect of methyl salicylate. This may be due to these compounds binding to the same binding pocket, affecting the cation channel. Eucalyptol acts as an inverse agonist by shifting the receptor's equilibrium toward the closed state, thereby reducing spontaneous activity [69]. There is also evidence indicating that these compounds are not merely inverse agonists, but instead act as negative allosteric modulators to influence the function of the receptor [126] (Fig. 3). This mechanism reveals a novel peripheral signal transduction pathway within inhibitory ORs that suppresses mosquito olfaction, offering new insights for preventing mosquito bites and developing more effective repellents. Recent investigations have identified CquiOR27 as another receptor with dual current directions, capable of producing inverse currents in response to different compounds [127].

The suppression of olfactory receptor activity by repellents such as DEET and indole, along with novel mechanisms like those of CquiOR32 and CquiOR27, underscores the diverse ways repellents can disrupt mosquito olfaction. These findings provide a deeper understanding of the complex interactions between repellents and mosquito olfactory receptors, which can be leveraged to develop more effective strategies for preventing mosquito bites and controlling mosquito-borne diseases.

## Conclusions

Insect olfactory receptors play a crucial role in the transmission of vector-borne diseases by governing key mosquito behaviors such as host location, oviposition site selection, and threat avoidance. Their adaptability to diverse environments highlights their significance in the ecological and evolutionary dynamics of vector-borne diseases. Given that mosquitoes transmit pathogens through blood-feeding, the variable response of different mosquito species to specific odors, due to the specificity of their olfactory receptors, significantly impacts host preferences and disease distribution. Thus, elucidating the structure, function, and odorant responses of olfactory receptors is essential for understanding disease transmission patterns and informing the development of novel drugs. The design of targeted chemical agents, such as repellents and attractants, which can modulate mosquito olfactory signaling, offers a promising avenue for controlling mosquito populations and behaviors, thereby aiding in disease prevention. Additionally, research on olfactory receptors is vital for devising new technologies to monitor mosquito populations and assess disease transmission risks. By targeting olfactory mechanisms, we can refine vector control strategies and enhance public health outcomes in the fight against mosquito-borne diseases.

## Data Availability

Data supporting the main conclusions of this study are included in the manuscript.

## Abbreviations

LLINs: Long-lasting insecticidal nets
IRS: Indoor residual spraying
ORs: Odorant receptors
ORNs: Olfactory receptor neurons
AL: Antennal lobe
PNs: Projection neurons
LNs: Local neurons
GRs: Gustatory receptors
IRs: Ionotropic receptors
OBPs: Odorant-binding proteins
AC: Adenylate cyclase
Orco: Odorant receptor co-receptor
CaM: Calmodulin
PKC: Protein kinase C
*An. gambiae*: *Anopheles gambiae*
*Ae. aegypti*: *Aedes aegypti*
*Cx. quinquefasciatus*: *Culex quinquefasciatus*
*Tx. amboinensis*: *Toxorhynchites amboinensis*
*Ae. albopictus*: *Aedes albopictus*
GPCRs: G protein-coupled receptors
cAMP: Cyclic adenosine monophosphate
DEET: *N*,*N*-Diethyl-3-methylbenzamide
